# Leisure Adjustments of Older Finnish Adults during the Early Stages of the COVID-19 Pandemic

**DOI:** 10.1007/s41978-022-00113-x

**Published:** 2022-07-06

**Authors:** Veera K. Koskinen, Emilia A. Leinonen

**Affiliations:** 1 Faculty of Social Sciences, University of Helsinki, Helsinki, Finland; 2 Faculty of Humanities and Social Sciences, University of Jyväskylä, Jyväskylä, Finland

**Keywords:** Late adulthood, Leisure, Physical distancing, Active aging, COVID-19

## Abstract

The current approach to “aging well” emphasizes the importance of active leisure participation in late adulthood. This relates to the view that leisure activities enable older adults to stay physically, mentally, and socially active, and they thereby contribute to wellbeing. In spring 2020, leisure activity engagement was significantly hampered by the COVID-19 pandemic. This study explores leisure-related experiences and adaptation strategies among Finnish older adults during the period of strict physical distancing. The data comprises letters (N = 77) written by Finnish people (aged 70–93) that were analyzed using content analysis. The study suggests that the reorganization of leisure was particularly influenced by the social significance of leisure activities, the age identities of the participants, and the prevailing ideals of active aging. The paper introduces three strategies of leisure adjustment: building new routines, maintaining activeness, and enjoying slow leisure. The article highlights the importance of investing in older adults’ leisure participation in the aftercare of the pandemic.

## Introduction

Over the past few decades, research interest in the leisure involvement of older adults has grown considerably. As an increasing number of people can expect to live into their sixties and beyond in relatively good condition, the lifestyles of older adults have become more active and leisure-oriented (Karisto, 2007; Katz, 2005). Many older adults with regular involvement in leisure activities also report positive wellbeing, which has further stimulated discussion on the benefits of leisure participation in late adulthood (Gibson & Singleton, 2012; Nimrod, 2007). The positive connection between involvement in leisure activities and the wellbeing of older adults is especially emphasized by the so-called activity theory of aging (Havighurst, 1963) and its refinements (e.g. Rowe & Kahn 1997). Prior research widely suggests that, in a stage of life with fewer responsibilities related to a career and parenthood, involvement in leisure activities may have an important compensating purpose for older adults by providing them opportunities for social interaction, self-expression, and self-development (e.g. Foster & Walker 2014; Gibson & Singleton, 2012; Nimrod, 2007; Van der Meer, 2008).

Encouraging older adults to participate in leisure activities also forms the foundation of the active aging strategy in many countries (WHO, 2016). Keeping the older population physically active and socially involved is viewed as one way of mitigating the future costs of care. Previous research has noted that the activity-oriented approach to aging widely dominates the public debate and commercial imagery on aging and forms also the background for older adults’ own reflection on aging and old age (e.g. Lumme-Sandt 2011). Especially in Western countries, the competence of older adults is increasingly measured by their ability to lead an active lifestyle that involves social engagement and healthy lifestyle choices (Katz, 2005). In this regard, active leisure participation after the withdrawal from working life appears as a legitimate strategy for older adults to respond to the prevailing ideals of aging, i.e. “the busy ethic” of aging (Katz, 2000).

In spring 2020, leisure activity engagement was significantly hampered by the outbreak of the global pandemic that was caused by the coronavirus (COVID-19). In Finland, a recommendation was issued for people over 70 years of age to stay in “quarantine-like conditions” or at least to avoid meeting others in person (Government of Finland, 19.3.2020). The purpose of this recommendation was to protect those at a higher risk of having serious complications due to the coronavirus as well as to secure the capacity of hospitals. As there were almost 900 000 people aged 70 or over in Finland at the end of 2019, which comprises over 15% of the whole Finnish population, the age-based preventative measure had broad implications on the lives of many older adults and their families (Statistics Finland, 2020a). Although it was generally seen as important to protect older people, the recommendation also sparked a critical debate from the perspective of individual freedom (Lumme-Sandt et al., 2020).

By combining sociological discussions on late adulthood with leisure research, this article aims to identify leisure-related adaptation strategies among older Finnish adults during the first wave of the COVID-19 pandemic. In this paper, leisure is broadly defined as time that is free from obligations, i.e., time during which it is possible to engage in un-coerced and personally valued activities (e.g. Kleiber 1999). In the Finnish media, there has been a lively discussion on how social distancing has impacted the leisure activities of the working aged population (e.g. YLE, 2021; MTV news, 2021). For instance, it has been highlighted that people have begun to make more use of local recreational facilities, spend more time on digital leisure, and even re-consider their own place of residence due to the commonization of remote working. However, little attention has been paid to how the leisure practices of the older adults changed after the outbreak of the pandemic. Inregarding aging and retirement (Korn the case of older adults, the discussion on the impacts of physical distancing has mainly focused on aspects of health and care, such as the potential decline in old people’s functional capacity, increased feelings of loneliness and access to care (e.g. Atzendorf & Gruber 2021; Aaltonen et al., 2021; Paananen et al., 2021).

Overall, the perspective of vulnerability is often emphasized in societal debates that concern the life of older adults during the COVID-19 pandemic. Little is known about how relatively healthy and independent older adults with varying personal resources and situations in life adjusted to this unusual situation. Nonetheless, many older adults continue to work, volunteer, travel, engage in hobbies, and remain otherwise active participants in society, and they necessarily do not consider themselves to be in need of extra protection and care. It is also well known that, growing older is not only about physiological deterioration, but an individual’s psychological well-being, including positive thinking, balance of emotions, and overall satisfaction with life, typically increases with age (e.g. Blanchflower & Oswald 2008; Carstensen et al., 2011). These qualities may also turn out to be an advantage in the event of a crisis such as a pandemic (Tiilikainen et al., 2021; Van Winkle et al., 2021).

In order to understand the diverse social implications of the COVID-19 pandemic, it is important to pay attention to the experiences of people at different ages and stages in life. This study contributes to this issue by providing insights on how the quarantine-like conditions affected the leisure practices of older Finnish adults. The study focuses on how the participants reacted to the preventative measures that greatly impacted their leisure practices and what kind of strategies they adopted to cope with the “new normal,” within the context of leisure.

## Literature review

### Perspectives on late adulthood

Since people are living longer and healthier lives, the period between middle age and old age has become significantly longer. In aging studies, these “extra years of life” are generally called the third age. This stage of life is conceptualized as an active period in late adulthood in which a person has relatively little age-related constraints but typically has more leisure time on account of a decrease in responsibilities related to family and career (e.g. Gilleard & Higgs 2007). Thus, the third age is widely denoted as a stage of life for self-actualization, purposeful engagement and active leisure (e.g. Karisto 2007; Katz, 2013). The time of physical and psychosocial deterioration and increasing dependence on other people’s support is considered to signify entry into the fourth age (Higgs & Gilleard, 2014). Although it is not possible to define a certain age at which the third age begins or when it turns into the fourth age, these conceptualizations are useful for discussing the prevailing norms and ideals that are associated with aging and late adulthood. Where the third age is portrayed as an opportunity to pursue personal goals and enjoy increased leisure time, the more negative connotations of aging have accumulated into the portrayal of the last years of life. That is, the fourth age has begun to be viewed as a less desired stage of life, the transition to which should be postponed as far as possible (Higgs & Gilleard, 2014; Kydd et al., 2018).

A number of scholars have pointed out that the current debate on “aging well” is increasingly dominated by the view that the individual’s own efforts and lifestyle choices determine how the aging process progresses and how late adulthood is experienced (e.g. Katz 2000, 2005; Settersten & Angel, 2011). It has been argued that, in the present society that is obsessed with performance, older adults are required to prove their competence and validate their retirement years by continued social engagement and consumerist lifestyles (e.g. Gilleard & Higgs 2007; Katz, 2000). The ideal of “active aging” becomes evident, for example, in media imagery that emphasizes lifestyle optimization and self-care as prerequisites for good aging (Lumme-Sandt, 2011). More recent studies have demonstrated the prevalence of this kind of thinking also in older adults’ own plans and wishes regarding aging and retirement (Kornadt & Rothermund, 2014; Koskinen et al., 2017; Marhánkova, 2011).

The concept of age identity is helpful in illustrating the individual’s subjective awareness of age, which does not necessarily coincide with the individual’s actual age. There is a wide consensus among social scientists that people do not automatically take up the role of an “old person” once they reach a certain age, but they strive to maintain consistency in their lives and identities. A combination of factors, such as gender, health, socioeconomic status and the surrounding culture, has an impact on the individual’s understanding of the normative course of life and aging, which, in turn, has an impact on whether a person experiences themselves as younger or older than their chronological age (Howard, 2000; Schafer & Shippee, 2010). In Western societies, where youthfulness is highly valued, older adults with younger age identities have been found to be more optimistic about their abilities and aging (Schafer & Shippee, 2010).

Comparing one’s own physical competence and lifestyle to that of others is shown to be a common strategy in developing and refining age identity in late adulthood (e.g. Kydd, 2018; Pietilä et al., 2013; Koskinen et al., 2017). People also tend to adopt social roles and lifestyles that other people and society reinforce through their actions and definitions. Thus, an individual’s perception of their own age and aging is, above all, a social construct. Previous research indicates that older adults more often associate old age with a loss of independence than with chronological age or health status (e.g. Heikkinen, 2000). This relates to the fact that today’s older adults have notably better physical and cognitive capacities than older adults that lived a few decades ago (Koivunen et al., 2020; Munukka et al., 2021). Moreover, since many chronic diseases have become manageable and do not necessarily significantly reduce an individual’s quality of life, the role of lifestyle in determining the individual’s social roles and age identity has increased in importance.

### Wellbeing and leisure in late adulthood

Aging is often approached from the viewpoint of loss both in terms of physical capacity and social roles, but late adulthood, as any other stage in life, also involves growth and development. For instance, getting older has been shown to strengthen the individual’s self-esteem and subjective wellbeing as well as to improve their emotional self-regulation (e.g. Blanchflower & Oswald 2008; Carstensen et al., 2011). Such psychological development may increase the individual’s adaptability and flexibility in daily life and help them cope not only with the changes that accompany aging but with a variety of other challenges in life as well.

Leisure activities may be an important way to relax and acquire new skills at any stage of life, but late adulthood entails specific life events and circumstances that may increase the importance of leisure participation. For example, the transition to retirement, the increased probability of contracting chronic diseases, and other physical and social changes related to aging typically change social relationships and the structure of everyday life. It has been suggested that participation in leisure activities may help older adults to adapt to these changes (e.g. Rowe & Kahn 1997; Genoe et al., 2018). The key is not necessarily the number of activities but rather their quality. For example, Nimrod (2007) found that personally valued and/or socially important leisure activities are the most beneficial for the subjective wellbeing of recently retired older adults. Some studies even suggest that leisure activities may serve as a tool to resist social norms and stereotypes related to aging and old age (e.g. Dionigi 2002).

Discussions on the benefits of leisure engagement in late adulthood often highlight more active forms of leisure. A variety of studies have established that physical activity has several positive effects on the functional capacity and health of older adults (e.g. Kekäläinen 2019). In addition, mental efforts and a flow state are shown to be beneficial for individual’s overall wellbeing (Stebbins, 2007; Stuart, 2022). In the case of older adults, it has been demonstrated that activities such as tourism (e.g. Hung & Lu 2016; Nimrod & Rotem, 2010), gardening (e.g. Wang & MacMillan 2013), volunteering (e.g. Cattan et al., 2011), and various group activities (e.g. Cooper & Thomas 2002; Stuart, 2022) include elements that support the physical, mental and social well-being of older adults. It has also been suggested that these kinds of activities offer possibilities for older adults to experience feelings of mastery, autonomy, and social connectedness, the attainment of which may have previously been linked to other domains of life, such as work (Gibson & Singleton, 2012; Stuart, 2022).

In recent years, there has been growing scholarly interest in going beyond the activity-oriented discussions on aging. An increasing number of studies underline that, while older adults might choose less physically active leisure activities with age, such activities are not necessarily less beneficial for their wellbeing (e.g. Adams et al., 2011; Standridge et al., 2020). As noted by Stebbins (2007), short lived, enjoyable experiences of casual leisure, such as watching tv or having coffee with a friend, are also necessary for the individual’s wellbeing. Furthermore, it has been illustrated that the benefits of leisure involvement are not necessarily related to the activity itself but to other aspects, such as an increased sense of togetherness and other positive emotions that rise from participating in the activity (e.g. Koskinen 2019; Stuart, 2022). In late adulthood, leisure activities also bear importance from the perspective of the individual’s everyday structures and routines (e.g. Miller 2016; Genoe, 2018).

In summary, it has been shown from this literature review that late adulthood creates a specific context within which to experience the pandemic and related preventative measures. This relates not only to the greater health risks to older adults but to the fact that the social relationships and meaningful life contents of many older adults are built around leisure activities, participation in which was hampered in many ways following the outbreak of the pandemic. Thus, it is important to pay attention to older adults’ pandemic related experiences precisely from the leisure perspective. This empirically driven study approaches the issue in the Finnish context by looking at how the Finnish participants over 70 years of age reacted the restrictive measures and how they re-organized their leisure routines and activities in spring-summer 2020.

## Materials and methods

Since at the time of the data collection in Finland all persons aged 70 or over were obliged to stay in quarantine-like conditions, a call for written letters was used as a data gathering method in order to reach older persons with diverse backgrounds from different regions. Finland is geographically large but sparsely populated country which meant that the restriction measures were implemented somewhat differently in different regions; for instance, in the beginning of the pandemic, most of the infections were concentrated in the capital region and for that reason, the whole region was isolated from the rest of the country (Forma et al., 2020).

The call for letters was open from early April to the end of June 2020. The writers were encouraged to write freely about their situation and day-to-day life after the outbreak of the pandemic. They were specifically asked how they had received the guidelines to stay in quarantine-like conditions. In addition, the writers were asked to report their age, gender, place of residence and to specify their family relations. No other demographic details were asked in order to keep the call as short and open as possible and to minimize the risk that potential writers would see the call as a structured questionnaire.

It was possible to participate anonymously unless the writer was willing to be contacted later for an interview. The writers could send their letters either by regular mail, by secured e-mail, or anonymously through the Webropol survey tool. The call was primarily shared on social media, on various regional Facebook pages of the largest cities in Finland in particular. Also, a bulletin was made which was published in four regional newspapers, which broadened the number of potential participants. The decision to share the call mainly on social media was made because of the relative high number of social media users in Finland. According to Statistics Finland (2020b), in 2020, 69% of persons aged 16–89 used social media, the most popular social networking site being Facebook. Of people aged from 65 to 74, over 300 000 persons were using social media; in the oldest group, that is, people aged from 75 to 89, the share was over 75 000 users.

In total, 77 letters were received (41 by e-mail, 20 by mail, and 16 by Webropol). The youngest writers were 70 and the oldest 93 years of age. Almost three quarters of the writers were women. About 60% were living with a spouse and 40% alone. The study has carefully followed the ethical directions of The Finnish National Board on Research Integrity. Informed consent was obtained by providing information on a website, which included data protection, the use of personal data, the rights of participants, and the purpose of the whole study. The privacy and data protection policy were also available as simplified text.

The received letters were analyzed by means of content analysis based on an interplay with previous research and observations that arose from the data (Krippendorff, 2012). The coding categories were derived directly from the text data, but the observations were interpreted considering previous studies on the life cycle transitions and leisure in late adulthood. First, all the letters (300 pages, the most typical length of one letter being 1.5 pages) were read through in order to form an overall picture of the collected data. After that, careful coding of the data was carried out to trace and connect similar pieces of information together. In the process of “thematic coding” (Gibbs, 2007), attention was paid to the points of data in which the respondents described their leisure practices either before or after the outbreak of the pandemic.

The further analysis, i.e. the intensive reading of the coded data, enabled the formation of final themes that depict the leisure-related experiences and leisure adjustments of the research participants. The first part of the analysis focuses on the overall picture of how the measures taken to prevent the spread of the pandemic were received by the participants and how the measures affected the participants’ leisure. The second part of the analysis presents the leisure-related coping strategies through which the participants adapted to the unusual situation. More precisely, the analysis seeks to answer the following questions:

1. How did the age-based preventative measures affect the participants’ leisure?
2. What kind of leisure-related adaptations did the participants perform during the early stages of the pandemic?

## Leisure-related interruptions

### When everything Changed

The received letters contain varying descriptions and views of the pandemic and its effects on people’s everyday lives. However, since the data was collected at an early stage of the pandemic, the experiences of sudden change and uncertainty about the future are recurring themes from one writing to another. As described in one letter:

*”But the strangeness has settled in. The first thing you remember in the morning is that the world is not like it used to be.” W72*.

The letters contain much information about how everyday chores, such as shopping, were arranged, but changes and re-organizations related to leisure are also prominently featured in the data. In fact, the cancellation of organized leisure activities, such as activity clubs, sporting activities, and cultural events, is quite often mentioned as a turning point by which the seriousness of the situation was realized and which gave a tangible form to the outbreak of the pandemic. As one of the participants wrote:

*”It was a Sunday night, and everything was fine. I went to [the name of the place] to dance tango. [There were] such good dance partners, and [it was] such a great evening, I looked forward to the next Sunday and [to the] slow waltz. -- Suddenly, everything stopped? And there I was, empty-handed, wondering. How to re-build the week? -- it feels like they are stealing time from me – and from other older people as well? I know our life won’t ever be the same! There’s not enough time.” W93*.

The excerpt above describes the sudden disruption in the habitual rhythm of life that, consequently, raised concerns about the near future. There is a sense of confusion and concern in the writing, but most importantly, it includes a solution-oriented reflection on how to cope with the situation and how to re-build one’s weekly routines. The excerpt also reveals the uncertainty associated with the new situation that does not arise only from the threat of the disease but more broadly from the reflection on one’s own age, one’s stage in life, and the meanings attached to the cancelled activities. That is, the crisis led to thoughts about the inevitable decline that is related to the aging process and to the possibility that one’s own life will necessarily never be the same again.

It is worth noting that also those participants whose everyday life was rather home-centered even before the pandemic expressed a lingering sense of dissatisfaction over not being able to physically participate in organized leisure activities. In the following excerpt, one participant, who is also a caregiver for his chronically ill wife, depicts his view on the importance of having activities outside the home:

*”Weekdays and holidays have become more and more indistinguishable. Sometimes you have to stop and think about the day of the week. After all, the days of the week have not been very important to us retirees before either, but there were milestones that kept life in a rhythm. On Tuesdays, I attended a writers’ group, on Wednesdays I took my wife to handicrafts, and in the evening, there was my woodworking group.” M71*.

The activity clubs mentioned by this man are examples of participatory forms of leisure the importance of which is generally emphasized in activity-oriented approaches to aging well. Nevertheless, the letters analyzed for this research illustrate that any leisure activity that is outside the home may bear particular significance in terms of daily routine, meaningful encounters, and experiences (see also Tiilikainen et al., 2021). Activities such as travelling, cultural events, and outdoor activities were described as activities that brought desired rhythm and variety into the participants’ lives. Therefore, it was also particularly difficult to leave such activities aside when the preventative measures were introduced.

### Falling into a Risk Group

Many of the letters include discussion and even anxiety about what the everyday lives of the writers will be like without their accustomed leisure activities. This contemplation is often connected to the disappearance of a daily routine, but much thought is also given to this theme from the perspective of health and wellbeing. The absence of some of the leisure activities made it more difficult to implement self-care and raised concerns about the maintenance of functional capacity and mental wellness. One participant stated:

*”I go to the gym regularly; I have never exercised at home. I was so irritated that I got angry, mad as a hornet, when the quarantine started, when just because of my age, without any chronic illnesses, I had to stop going to the gym. I am fearful of what is going to happen to my muscles, what is going to happen to me.” W73*.

Not being able to go to the gym frustrated this woman for two reasons. On the one hand, she finds it unfair that she had to give up this personally important activity only because of her age. On the other hand, she is afraid of the reduction in her functional capacity. She discusses both aspects in parallel with future-oriented fears regarding her own aging and wellbeing. This is noticeable in other letters as well: adequate functional capacity is not only thought to enhance one’s personal quality of life, but being in good condition more broadly exemplifies the participants’ capabilities and competence as “an aging person.” In this regard, the measures taken to prevent the spread of the pandemic appeared as an obvious threat to the well-being of the participants and also to their self-image and age identity. This can be seen to reflect the participants’ views on a satisfactory life and the societal ideals regarding aging and appropriate senior lifestyles (e.g. Katz 2000; Lumme-Sandt, 2011).

In addition to functional capacity, the individual’s lifestyle, i.e., the things that a person usually does, has an impact on their self-image and aging experience. Many of the participants described themselves as relatively healthy and active, and on the basis on their descriptions of their leisure practices, their life seemed rather independent and self-sufficient. Against this background, it was hard for some of the participants to digest the age-specific preventative measures.

*”—one beautiful winter morning, I suddenly woke up as an old person who belongs to a group of vulnerable people confined to quarantine-like conditions. At least it felt like that even though I have always been healthy. I take hardly any medications. I exercise, I take care of myself and so does my 73-year-old husband.” W70*.

As seen here, the frustration of this woman had nothing to do with an underestimation of the seriousness of the situation, but it rather arose from the fact that she perceived the preventative measures as contradictory to her self-image: how she saw herself, her own abilities, and her own lifestyle. Overall, the participants tended to connect the loss of independence with the fourth age in which physical and cognitive functional capacity has already clearly deteriorated. Because of this contradiction, some of the participants even perceived the age-specific recommendations as age-related discrimination.

## Leisure-related adaptations

### Building New Routines

In general, the received letters illustrate relatively calm and flexible attitudes towards the changed situation. Probably because of the participants age and life experience, they were able to realistically assess the situation, adapt to it, and even find some positive aspects in it. Focusing on one’s own routines proved to be a key coping strategy among the participants, especially in the early stages of the pandemic. One of the participants commented:

*” Although I still criticize the humiliating patronage of older people, I cope with these exceptional circumstances more easily than many younger people and young families. I get a pension, I can take care of myself, and [at this stage in life] staying at home doing nothing is totally acceptable. I hold on to my routine, I cook, I clean the house weekly, and I do laundry at a normal pace.” W73*.

Overall, the letters contain long and detailed descriptions of the participants’ daily activities, which indicates that planning and maintaining a new routine brought a sense of security under the exceptional circumstances. Some were even more concerned about how others were coping than how they themselves were doing. The writings show empathy especially towards the working age population and older adults who had a reduced functional capacity. Consequently, phone calls to family members and friends as well as to people with whom the participants had not been in contact for a while were part of the daily routine of many participants. Turning the focus on other people may have helped the participants distance themselves from their own situation and the negative feelings related to it, but it probably also helped address the situation better as a result of shared experiences.

As before the pandemic, the same personal interests guided the respondents’ leisure practices after the pandemic began. Many seemed to enjoy outdoor recreation, socializing, and self-improvement, and they displayed flexibility and creativity in their efforts to implement these interests in their new routines (see also Tiilikainen et al., 2021). The following excerpt is a good description of the importance of life experience in adapting to the new situation and of the writer’s willingness to continue her life “as usual”.

*“I followed the guidelines of my earlier crises: take care of yourself, go get a massage, remember to go to the hairdresser, eat properly according to your regular rhythm, take care of your sleep, use a bright light therapy lamp, don’t listen to everyone’s worries, quit watching the news, read a lot, be physically active, and learn to exercise at home. Online exercise classes saved me.” W73*.

Making more use of new digital technologies was also an important means for many participants in adjusting to the new normal and taking up new routines (see also Sivan 2020; Brooke & Clark, 2020; Reine et al., 2021). As noted by the woman in this excerpt, digital technologies enabled not only social interaction but also the continuation of some personally valued leisure activities. In general, the letters show that the use of mobile phones, the internet, and social media was part of the lives of many participants already before the pandemic. In addition, the same upward trend in the use of digital technologies for leisure purposes that is seen in the younger population is evident in the present data as well.

### Maintaining activeness

The letters mainly reflect the immediate reactions and changes in daily life after the outbreak of the pandemic. This temporal context partly explains the participants’ eagerness to keep themselves “busy” and take up new activities. Some of the participants themselves noted that some of their actions, such as intense cleaning of the house, long walks, or the rapid completion of unfinished handcrafts, were adaptation mechanisms to deal with the sudden change (see also Brooke & Clark 2020). As in the following excerpt, activeness also emerges in some of the writings as a means to create a diversion from the news and worries related to pandemic.

*”At some point, I noticed a need to distance myself from the corona overload. Otherwise, I would lose my mind. [I turned the] TV off, and [allowed myself] to read only a little bit of corona-related news. I started spending more time outdoors.” W73*.

Outdoor activities were an important part of many participants’ lives even before the pandemic, but their significance was further emphasized during physical distancing. In a couple of letters, walking and being outdoors are described as a way of passing time, but most respondents discuss these activities in relation to their efforts to maintain activeness and overall wellbeing (see also Tiilikainen et al., 2021). As one participant wrote:

*“Either I go alone or we go together for a walk near the beach and in the woods almost every day! It provides sufficient physical effort, [space for] thinking, and then things become clearer -- indoors [I have done] stretching and exercising as often as possible.” W70*.

While describing their physical activities, the writers tend to use expressions such as “as much as possible” or “as often as possible” to highlight the importance of engaging in this type of activity on a regular basis. A significant number of participants mention either the duration or the target length of their daily walks, which can be interpreted as one way of emphasizing their willingness and efforts to maintain physical activeness. In addition, the letters indicate a willingness to support the activity and wellbeing of others. For example, one of the participants, an 86-year-old man, had agreed to go on regular walks with his neighbor, who was in worse physical condition than he was. Correspondingly, in the following excerpt, one participant describes how she has been organizing outdoor activities jointly with others.

*”I have taken walks with a friend in the mornings. Then I figured that we could organize outdoor exercises here since we have this big yard. There were 3–8 of us grannies there [exercising]. Now that it is hot, we haven’t been able to exercise. [Instead,] we have been swimming, [and we have done] water running 2–3 times a day.” W71*.

In addition to keeping the body on the move, many also strived to keep their minds active. Activities such as writing, studying, and handicrafts are mentioned as new or rediscovered activities that have provided entertainment and opportunities for self-improvement at home.

*”-- I’ve started painting after a few decades of pause. -- as a new hobby I’ve also started making videos. I make 1–2 short videos on my Facebook page every week about my life or any other interesting topic.” M72*.

Although some of the letters reflect the strains and boredom caused by physical distancing, for some, as for the man in this excerpt, the situation seemed to nurture individual strengths and creativity. Similar experiences of increased creativeness can certainly be found in all age groups. However, for retirees, for whom leisure activities may be the most important mean of self-expression and self-development, the ability to develop substitutes for lost activities proved to be a particularly effective way of adapting to quarantine-like conditions.

### Enjoying slow leisure

As for younger generations, the new normal also increasingly directed the participants’ attention to forms of leisure that are more passive or slow in nature (see also Breunig 2020). The time that was spent on social activities, hobbies, events, and trips before the pandemic was replaced by slow, nature-focused leisure activities and tranquil indoor activities. Most of the participants mentioned that they had watched television, listened to the radio, and read books more than before the pandemic. As was written by one of the participants:

*“Now has been a good time to watch that television series that I have not previously seen as well as my old [television series] favorites. Almost every day, we [I and my husband] go for a walk with walking poles -- I also have small projects going on all the time, [and] we try to write down old things [that happened in our lives] now that we still remember them. Rainy days are really good for that.” W70*.

Experiences of boredom and passivity were also reported, but not everyone felt that the change was for the worse (see also van Winkle et al., 2021). Instead, quite many highlighted how the relaxation of the pace of life had a calming effect on the mind and made it possible to focus on the most essential things in life. For example, a 73-year-old woman who still worked part time before the pandemic noted that she is now really “enjoying retirement for the first time.” Similarly, some felt that the end of scheduled leisure activities gave room to think about the past and the future and helped them realize the good things in life. Some of the participants, such as the woman quoted below, had lived a life filled with voluntary work and hobbies before the pandemic, but now they enjoyed the calmer rhythm of life.

*”For the first time in my life, I can do the things I like without a bad conscience or the need to explain anything. Incredible feeling!” W70*.

Regarding the re-organization of leisure, the responses of younger participants in particular demonstrate the same kind of positive experiences that have been reported among working aged people. For example, they write that they have begun to enjoy being at home and spending more time with their partner. In general, several respondents expressed that they no longer want to fill their calendar with that many leisure activities after life returns to normal.

One interesting feature of the letters is that they contain a considerable amount of descriptions of nature, animals, and changes of seasons that, in a rather charming way, reflects a “return to the basics” due to the restrictive measures. Some even mention that they now pay more attention to nature and spend more time observing it. In addition, new ways of spending time with friends and relatives were created in outdoor spaces.

*“My only activity outside the home is walking in an area of a few blocks. During these walks, I have been meeting my friends, too. They belong to the risk group as well. We take turns in who offers a cup of coffee and buns as we visit the graves of our husbands.” W81*.

As shown in the above example, proximity to nature and the possibility to meet people outdoors was an important source of wellbeing for many. On the other hand, although the refreshing and empowering benefits of nature-focused leisure are highlighted in several letters, it is also evident that enjoying outdoor activities was much easier for those participants who had their own yard or summer cottage or who lived in areas with good opportunities for outdoor recreation.

## Discussion

The present study was conducted to take part in the topical debate on the social implications of the global COVID-19 pandemic from the perspective of late adulthood. By looking into the pandemic-related writings of Finnish adults aged 70–93, this study has focused on changes in the leisure practices of older adults after the Government of Finland recommended that people over 70 years of age should stay in “quarantine-like conditions.” We examined how the participants responded and adapted to this new situation in the context of leisure. The aim was to shed light on the leisure involvement of older adults both during the pre-pandemic period and during the first few months of the pandemic. This perspective provided the opportunity to discuss not only specific impacts that the pandemic had on the lives of the participants but also the impact of the pandemic on prevalent ideals regarding aging and late adulthood.

As outlined at the beginning of the paper, longevity has significantly increased the opportunity of older adults to contribute to society and pursue personal goals outside the context of paid work (e.g. Gilleard & Higgs 2007). Simultaneously, “senior lifestyles” are now more dynamic and framed by activities and consumption practices that enable the expression of one’s individual values and identity (e.g. Katz 2000, 2005). Considering the participants in this study, the clear majority of whom were still living relatively independent and active lives in their retirement years, the recommendation to stay home and avoid meeting others in person significantly narrowed their opportunities to engage in the social roles and activities that they preferred.

Even though the letters received for this research display considerable variation regarding the leisure-related interests and activities of older adults, the present findings support the notion that having leisure activities outside the home is an important means for older adults to be socially engaged and construct their own sources of purpose, fulfillment, and well-being in late adulthood (e.g. Gibson & Singleton 2012; Nimrod, 2007). Unlike, for example, in the case of employees or students, whose “main responsibilities” (working and studying) remained more or less the same despite the restrictions regarding leisure participation, the writings of older adults reflect a situation in which the changes in leisure in particular had an impact on the rhythm and structure of their everyday life. Accordingly, the situation required adaptation strategies that specifically targeted the reorganization of leisure.

The results indicate that, for some older adults, adapting to a “new normal” was challenging from the viewpoint of age identity, which is a person’s subjective, yet socially constructed, awareness of their own age and the key social roles related to it (Schafer & Shippee, 2010). Due to the introduced preventative measures, those older adults that considered themselves to be relatively healthy, active, and independent found themselves in a situation in which they were treated as vulnerable people in need of protection. In the context of leisure, this conflict over age identity was found particularly challenging due to uncertainties related to the duration of the pandemic and one’s own stage in life. That is, the participants feared that “the golden years of the active third age” would be spent in quarantine-like conditions.

According to our findings, *building new routines* was the most immediate way of responding to changed circumstances. Focusing on everyday structures seemed to aid in maintaining continuity, meaning, and security in the participants’ lives despite the great uncertainties. As noted in the introduction, older adults are often considered a vulnerable group in societal debates and regulations. However, in the light of our results, in the event of a crisis, older age and life experience can also turn out to be a strength. Precisely because of the participants’ age, they had already experienced various kinds of hardships in their lives and thus accumulated individual knowledge and coping mechanisms that helped them adapt to the loss of accustomed routines. It is also important to note the importance of the retirement and the Finnish pension system in terms of financial security: unlike many working age adults, older adults aged 70 or more were not financially impacted by the pandemic (see e.g. Sihto et al., 2022). In addition, although the concern had been raised that replacing face-to-face meetings with digital technology may be more challenging for the older population, the participants in this study seemed to be motivated to make more use of digital information technologies to maintain social relations and to continue personally meaningful activities (see also Tiilikainen et al., 2021; Chung et al., 2021).

Another common adaptation strategy identified in this study was the *maintaining of activeness*, which refers to a variety of activities that the participants adopted to keep themselves mentally and physically busy. These activities involved, for example, learning new skills, keeping up physical activity, and immersing in a variety of leisure-related projects. Regardless of one’s age and stage in life, keeping oneself busy is a natural way to respond to a difficult situation. However, our findings illustrate that the participants were also motivated in their efforts to maintain the kind of lifestyle they were accustomed to during pre-pandemic times. Maintaining activeness seemed to help in tackling feelings of uselessness that some felt when they could not take part in personally meaningful tasks and activities.

The present findings provide indications that, for some older adults, health-related control became accentuated when participation in organized activities was restricted. This resulted, for example, in greater involvement in self-organized activities related to exercising and self-care. In none of the letters, however, was the maintenance of a good physical condition discussed from the viewpoint that it could prevent the participants from getting severely ill from COVID-19. Rather, intensive self-care appeared as a means of dealing with the changes and uncertainty of everyday life and as a means of keeping oneself healthy “for the better days to come.” As the societal debate on aging increasingly concentrates on the functional capacity of older adults, that is, what a person can do and how effectively, this kind of thinking seemed to be reflected in the writings and lifestyle choices of the participants in this research as well.

During the pandemic, increased significance was also given to *slow leisure*, by which we refer to activities such as observing nature and listening to music. The finding is well accord with recent studies that demonstrate that spending time outdoors may allay some of the difficult emotions that arise in the event of a crisis and may therefore serve as an important tool to adjust to atypical circumstances, such as physical distancing (Breunig, 2020; Brymer et al., 2021; Lackey et al., 2021). The present findings also indicate that the local environment, such as green areas, walkways, and recreational places in the vicinity of one’s home, became important meeting places and offered desired variation for home-based leisure (see also Tiilikainen et al., 2021).

The participants’ descriptions of slow leisure also revealed interesting contradictions between the current ideal of active aging and older adults’ own views regarding a personally satisfying lifestyle. The opportunity to slow down one’s rhythm of life due to cancelled activities was perceived to be beneficial in evaluating one’s own accustomed routines and identifying the activities that the participants wanted to prioritize after the situation returns to normal. For some, this reflection even led to the determination to spend less time on scheduled activities. As noticed in the literature review, active forms of leisure are often considered superior to more passive forms of leisure, especially in the context of aging. In this regard, the findings of this study provide an interesting view on how quarantine-like conditions provided a change for some older adults to experience a more simple and serene life without feeling the guilt of being useless or unproductive. Thus, it can also be assumed that slow leisure and/or spending leisure time more at home may continue to be a personally preferred choice for some older adults also after the pandemic.

Although the leisure-related observations obtained in this study offer an important counter perspective to the general debate on the vulnerability of older adults in the context of the pandemic, it is worth recalling that the resources and possibilities for refreshing and meaningful leisure were not the same for all of the participants, not to mention all older adults. Not everyone had a big yard in which to do gardening, a partner with whom to spend time indoors, or adequate digital skills to participate in online exercise classes. It is also well known that the loneliness, anxiety, and depression that some individuals experienced during the physical distancing make the mind more inflexible, arousing feelings of hopelessness instead of creativity and solution-oriented approaches to change. This was evident in some of the letters received for this research, although in the context of leisure, most of the participants seemed to adapt to the changed circumstances quite well. In general, it is important to note that the research involved people from different backgrounds, each of whom experienced the outbreak of the pandemic in an individual manner. In order to provide an overview of leisure-related changes and adaptation strategies, not every aspect of the data could be reflected in our final analysis.

Overall, the scope of this study was limited to the views of older adults who were motivated and had the possibility to send a letter. We can assume that the experiences of older adults with significant decline in physical and cognitive functioning and those who suffered the most due to the pandemic were excluded from this study. Although the study provides useful insights into how older adults reacted and adapted to age-specific restrictive measures, the findings of this study cannot be generalized to all Finns over 70 years of age. Therefore, further research with varying methods should be undertaken to understand what the implications of the pandemic are for the diverse older adult population.

In the case of older adults, concerns have already been raised about the decline of their functional capacity and an increase in their loneliness during the strict physical distancing (e.g. Atzendorf & Gruber 2021). Besides, the present findings point to the importance of investing in the social engagement and leisure activities of older adults in the aftercare of the pandemic. As discussed above, social gatherings and organized leisure activities are crucial in offering opportunities for older adults to interact and bond with others. By engaging in leisure activities outside the home, older adults do not only pursue better skills for “active and successful aging,” but most importantly, they create meaningful social roles that increase their sense of belonging to family, community, and society. While we can expect that some older adults will rather effortlessly return to their core leisure activities as soon as it is safe, some may need more encouragement and support - a matter that should be considered by the societal actors that provide aid and activities for older adults. Furthermore, even though almost all restrictions have already been lifted in Finland, it is evident that not all citizens feel that they can participate in activities safely. Therefore, societal actors should take into consideration how to ensure that risk groups such as older adults and persons with weakened immune defense could participate in leisure activities in the same way as others.

## Electronic supplementary material

Below is the link to the electronic supplementary material.

Supplementary Material 1
