# Supplementary material for: Leisure Adjustments of Older Finnish Adults during the Early Stages of the COVID-19 Pandemic
Source: Int J Sociol Leis. 2022 Jul 6;5(3):373–91. doi: 10.1007/s41978-022-00113-x (PMC9257554; doi:10.1007/s41978-022-00113-x)
Supplement: Supplementary file 1 — Supplementary Material 1 [file 41978_2022_113_MOESM1_ESM.docx]

| **Reviewer comment** | **Response** |
| --- | --- |
| **Reviewer #1**  I find the manuscript "Leisure Adjustments of Older Finnish Adults during the Early Stages of the COVID-19 Pandemic" very well-written, original, and in many ways a suitable publication for this journal. It is a unique contribution to the flourishing literature on the effects of the COVID-19 pandemic to cultural practices. | We would like to thank the reviewer for the supportive feedback and good suggestions. |
| I am recommending minor revisions, and all of my points of action can be identified in the text boxes above. They mainly have to do with contextualizing better the Finnish case and discussing the possible limitations of the data. I suggest that the very last paragraphs of the manuscript could be added some emphasis. In the aftermath of the pandemic, what will happen next in the cultural participation patterns of the elderly people in this relatively egalitarian context? Will some newly found patterns remain, will something go directly back to where they were before the pandemic? What are the implications of this for cultural policy and leisure in a more general way? | To better contextualize the study, we have now added more information on the Finnish-case (see. p. 8, lines 174-181, p. 20, lines 461-463).  We have now added more future-oriented reflection in the discussion (see p. 21, lines 502-504 and p. 23., lines 535-539) |
| **Reviewer #2**  This paper addresses an interesting and timely topic and is generally well-written. I have a few suggestions for improvement.  Page 2, lines 37-46 - please provide a citation for information about the pandemic restrictions. Similarly, please provide citations related to the media debate about social distancing mentioned on page 3, lines 13-20. | We would like to thank the reviewer for the supportive feedback and precise comments.  We added the required references (see p. 3, line 54). We also changed the word “debate” to “lively discussion” as it better describes the quality of discussion in the media (see p. 3, line 53). |
| Page 4, top of the page - it is not necessary to include methods details here that will be repeated in the methods section. This paragraph should very clearly state the purpose of the study. | We agreed and modified the paragraph. |
| Page 7 - the discussion of serious leisure didn't seem to fit well with the current study. It was not part of the purpose or methods. When mentioned in the findings section on page 10, there was no evidence that the activities mentioned fit with Stebbins' defining characteristics of serious leisure. I would suggest either developing this connection more thoroughly or deleting discussion of the concept | Using the concept of serious leisure in this context is, in our view, justified, but we agree that using the concept would have required broader discussion both in the analysis and in the literature review. Therefore, we decided to delete the discussion on the concept entirely as we didn’t have enough space to have more in-depth discussion on it in this paper. However, we think that the subject is worth discussing in the forthcoming articles based on our data. |
| The section on wellbeing and leisure in late adulthood could be a little bit more thorough in terms of reviewing the literature on leisure and aging as related to the purpose of the current study. One of the two research questions is focused on leisure-related adaptation, yet this doesn't seem to be a focus in the literature review. For example, Rebecca Genoe has done research on leisure and the transition to retirement. The following reference discusses the importance of developing routines as part of the adaptation process, which would fit well with the current study:  Genoe, M. R., Liechty, T., & Marston, H. R. (2018). Retirement transitions among baby boomers: Findings from an online qualitative study. Canadian Journal on Aging/La Revue canadienne du vieillissement, 37(4), 450-463. | Our second research question is the following: *What kind of leisure-related adaptations did the participants perform during the early stages of the pandemic?* (see p. 9, lines 220-221) Accordingly, our analysis focuses on leisure-related adaptations in the context of the pandemic, and therefore the introduction and literature review are constructed to provide an overview of the conditions within which the leisure-related changes of our study participants were made (the outbreak of pandemic and strict age-based restrictions in Finland).  With regard to our research questions, it was also necessary to provide an overview of the typical life cycle transitions in late adulthood, discuss the contemporary notion of “active ageing”, and provide information on some other issues influencing leisure in late adulthood. As noted in page 6, line 135, we recognize that transition into retirement is a significant life cycle transition affecting older adults’ leisure. However, taking into account the research questions of this study, we see no need to discuss adaptation to retirement (or other life cycle transitions) in more detail. Nevertheless, the article suggested by the reviewer (Genoe et al., 2018) fits to our literature review and citation to it has been added in the p. 6, line 138. |
| The jump from the end of the literature review to the beginning of the methods seems a little bit abrupt. It would help to have either a brief paragraph at the end of the literature review to summarize existing gaps in the literature and how the presented literature justifies/contextualizes the current study, or some of these thoughts woven throughout the literature review section. | We added a paragraph on how the presented literature contextualizes our study (p. 7, lines 164-172). |
| Page 8 - I thought it was interesting to use letters as the data source (as opposed to interviews or focus groups, for example). I can imagine pros and cons. It might be nice to add a sentence or two at the beginning of this page to explain why this approach was chosen. | This approach was selected in order to reach older people with varying backgrounds and from different regions. We added a sentence (p. 8, lines 174-176) to clarify why this method was chosen. |
| Page 8 - a few more details could be added including: a) how was the invitation was shared on social media? Through personal contacts? Older adult-related groups? b) what was the inclusion criteria for participants? The study seems to be focused on more independent/healthy older adults - was this screened for? c) Is there any more demographic data on the sample (e.g., race, income, education, work status)? | a) We added information on how the invitation was shared (mainly on regional Facebook pages), see p. 8, lines 190-192.  b) The only inclusion criterion was that the participant should be aged 70 or over, see p. 8, lines 174-176  c) Unfortunately there’s no more demographic data on the sample, but we added some general overview of the Finnish context, see. p. 8, lines 174-181, p. 20, lines 461-463 |
| Page 12, line 12 - I would suggest avoiding phrases like "it is generally known." If the information is from the data/findings, make that clear. If it is from the literature, cite the source. | We agreed and deleted the phrase. |
| Page 12, line 33 - this point about the participant not wanting to be lumped in with older adults would connect well with some of Rylee Dionigi's work on aging, sport, and the impact of neoliberal attitudes as those older adults who are active and healthy can tend to reinforce the blaming or othering of those who may not have the opportunity to be. | We added a reference to *Dionigi, R. A. (2002). Resistance and empowerment through leisure: The meaning of competitive sport participation to older adults. Loisir et Société/Society and Leisure, 25(2), 303-328.* |
| Page 14, line 56 - Tiilikainen et al., 2021 is referenced several time in-text, but not included on the reference page. | The article by Tiilikainen et al. is now included in the references. |
| Page 16, line 37 - The authors note "Experiences of boredom and passivity were also reported, but not everyone felt that the change was for the worse." Then they go on to provide details as to silver-lining type perspectives. I found it interesting that no quote or depth was provided for those more negative experiences. I wonder if this is because the majority of participants were on the positive side? Was this based on the purpose of the study? It might help to clarify the balance in the data between participants struggling with the data and adapting well/coping. | The sentence to which the reviewer is referring is placed in the section of our analysis in which we discuss how and why some of our participants came into the conclusion that they do not want to return to their accustomed leisure routines (full calendar, many participatory activities etc.) after the pandemic ends. In this context, it is relevant to write that not everyone felt that feeling bored or being more passive is a bad thing.  Our study focuses on how the preventative measures in Finland affected the participants’ leisure and how people adapted to these changed circumstances in the context of leisure. Viewed from this perspective, the "more negative" experiences did not emerge. In the revised version of our manuscript, we have clarified the perspective of our study (see p. 22, 511-516).  We had already noted (p. 22, lines 517-520) that the experiences of those who suffered the most due to the pandemic may have been excluded from our study because those people may have not have strenght/possibility/willingness to participate this study. In other words, we had already recognized this as a weakness of our study.  It was also already mentioned in the introduction and discussion (see p. 3, lines 58-65, p. 21-22, lines 504-511 and p. 22, lines 524-526) that concentrating on the more unpleasant consequences of the pandemic (e.g. increased loneliness, depressive symptoms etc.) is important, but also something that the previous studies have already dealt with. We also mention that our study serves a counter perspective to the general debate on the vulnerability of older adults in the context of the pandemic. We have now modified and added even more discussion on the perspective of our study, in order to avoid misunderstandings (see p. 22, lines 511-516).  In order to clarify the balance in our data between those participants who were mainly struggling and those who were mainly adapting we refined the phrase in the p. 22 lines 511-516. |
| Throughout the findings section, there could be a little bit more connection to other leisure research on the pandemic (which is understandably limited). For example, a special issue of the World Leisure Journal was published in 2021 focusing on COVID and aging. A paper that seemed related to the current findings is:  Chung, W., Rebecca Genoe, M., Tavilsup, P., Stearns, S., & Liechty, T. (2021). The ups and downs of older adults' leisure during the pandemic. World Leisure Journal, 63(3), 301-315. | We agree, and added reference to article by Chung et al. (2021), see p. 20, line 466. |
| The authors' approach to the findings section was to include some discussion and connection to existing literature throughout. I thought this was fine, but I was then a bit surprised to see such a long Discussion section. I would suggest shortening that section to be more of an overview and summary of the unique contribution to the literature. Some of the writing could be condensed and some could be moved into the findings section. | In the analysis we have added references to those parts of the text where there were clear links with the findings of previous studies. In the discussion, we wanted to build broader links with previous literature and discuss our findings from a wider perspective than only based on our own empirical data.  We agree that the discussion could have been a bit shorter, and therefore some parts of it have been deleted entirely from the revised manuscript. However, as the reviewers asked us to give more emphasis to certain issues that were included in the discussion, the length of the discussion did not change. |
| **Reviewer's Responses to Questions**  Is the presentation and style appropriate?  Reviewer #1: I find both presentation and style appropriate and with good scientific writing.  Reviewer #2: Generally, yes |  |
| How clear is the argument?  Reviewer #1: The argument – that the COVID-19 pandemic seriously affected Finnish “older adults” who were forced to restrict their pre-pandemic leisure activities and who then adapted different strategies of adjustment, coined here as “building new routines”, “maintaining activeness” and “enjoying slow leisure” – is extremely clear.  Reviewer #2: Fairly clear, but could be stronger |  |
| Does the abstract reflect the content?  Reviewer #1: The abstract covers very well the content. If there is any extra space in the abstract, I would perhaps add a sentence about how the empirical data were analysed.  Reviewer #2: Yes | We added a sentence about how our data were analyzed in the abstract. |
| What is the originality?  Reviewer #1: The originality of the paper lies without doubt in its use of empirical data. Data related to the effects on leisure caused by the COVID-19 epidemic is still scarce. In the case of this paper, the data does not only directly talk about the implications of the epidemic on leisure, it is also rather large in size. This could be perhaps pointed out as an extra asset or contribution of the paper.  Reviewer #2: The data is original and provides an interesting contribution to the literature. | - |
| Does the submission show awareness of related literature?  Reviewer #1: The submission definitely shows a good awareness of the related literature.  Reviewer #2: Somewhat - a bit more in terms of aging literature than leisure literature specifically. The depth of thought in terms of theory and connection to existing literature could be deeper | - |
| What is the methodological merit?  Reviewer #1: Like mentioned above, it is a good merit to have such a rich textual data – 77 letters written by a specific group. Even is there is already plenty of details about the data, I think it would be useful to specify how long the letters were (now it’s mentioned that there are 300 pages altogether, but perhaps a more concrete measure would be a mean length).  Reviewer #2: fine | We agree. The most typical length of a letter was about 1.5 pages (see p. 9, lines 206-207. |
| What is the theoretical importance of the submission?  Reviewer #1: I find that the theoretical importance is perhaps the weakest point of the paper – it is very descriptive and empirically oriented and does not depart from only one specific theoretical framework but points out several in the literature review. On the other hand, it does not claim to be a theoretically inclined paper, so I do not see a problem here. At most, I would perhaps mention that the paper is “empirical” or “empirically driven”.  Reviewer #2: provides insight into the impact of the pandemic on leisure, which is timely | We agree and point out in the revised version that our article is empirically driven (see p. 7, line 169). |
| What is the submission’s breadth of disciplinary interest?  Reviewer #1: The paper will be of particular interest for leisure scholars, but it will fall into the scope of interest of many sociologists as well.  Reviewer #2: connects well to aging and lesiure | - |
| What is the submission’s contribution to the area of study?  Reviewer #1: The study’s main contribution is that it provides yet another insight into how the pandemic has changed leisure structures. While the contribution is in this sense not entirely original (meaning that papers with similar points of view are published constantly), the empirical data is original. The paper could perhaps ponder more on how some of the changes found in the peoples leisure practices are perhaps more profound than others – it could be that some of the online activities mentioned could remain important even when the pandemic is finally “over” at least in the sense that the restrictions are lifted.  Reviewer #2: moderate | We have now added more future-oriented reflection in the discussion (see p. 21, lines 502-504 and p. 23., lines 535-539) |
| Discuss the breadth of geographical relevance of the submission.  Reviewer #1: The paper deals with a very limited geographical area – Finland. That is not a problem as such, because the paper does not claim that its findings are generalizable in any way. Meanwhile, the context could be discussed more than what currently is said. Now the country context is only specified in terms of the age structure and media use, little else. Could something be said about things such as class, education or especially equality? Many studies have pointed out that Finland is among the most equal or egalitarian countries in the world, which without doubt is also reflected into the leisure of people – for instance, it could be that elderly Finns are in a particularly good situation compared to their global counterparts (quality public healthcare, pension system etc.).  Reviewer #2: limited to Finland, but the pandemic is a global issue | We have now added more discussion on the Finnish case (see. p. 8, lines 174-181, p. 20, lines 461-463). |
| Discuss the suitability it for the journal.  Reviewer #1: I find that with some additional contextualization on the Finnish case, and perhaps a more thorough discussion of the possible limitations of the data, the paper suits the International Journal of the Sociology of Leisure very well and will find many interested readers.  Reviewer #2: good fit | We have added additional information on the Finnish case (see. p. 8, lines 174-181, p. 20, lines 461-463).  Limitations of our study are discussed in p. 21-22, lines 504-523. |
